# Endophytic *Diaporthe* Associated With *Citrus grandis* cv. Tomentosa in China

**DOI:** 10.3389/fmicb.2020.609387

**Published:** 2021-02-09

**Authors:** Zhangyong Dong, Ishara S. Manawasinghe, Yinghua Huang, Yongxin Shu, Alan J. L. Phillips, Asha J. Dissanayake, Kevin D. Hyde, Meimei Xiang, Mei Luo

**Affiliations:** ^1^Innovative Institute for Plant Health, Zhongkai University of Agriculture and Engineering, Guangzhou, China; ^2^Center of Excellence in Fungal Research, Mae Fah Luang University, Mueang Chiang Rai, Thailand; ^3^Faculdade de Ciências, Biosystems and Integrative Sciences Institute (BioISI), Universidade de Lisboa, Lisbon, Portugal; ^4^School of Life Sciences and Technology, University of Electronic Science and Technology of China, Chengdu, China

**Keywords:** nine new host records, two new species, *Diaporthales*, phylogeny, taxonomy

## Abstract

*Diaporthe* species are associated with *Citrus* as endophytes, pathogens, and saprobes worldwide. However, little is known about *Diaporthe* as endophytes in *Citrus grandis* in China. In this study, 24 endophytic *Diaporthe* isolates were obtained from cultivated *C. grandis* cv. “Tomentosa” in Huazhou, Guangdong Province in 2019. The nuclear ribosomal internal transcribed spacer (ITS), partial sequences of translation elongation factor 1-α (*tef1*), β-tubulin (*tub2*), and partial calmodulin (*cal*) gene regions were sequenced and employed to construct phylogenetic trees. Based on morphology and combined multigene phylogeny, eleven *Diaporthe* species were identified including two new species, *Diaporthe endocitricola* and *D. guangdongensis*. These are the first report of *D. apiculata*, *D. aquatica*, *D. arecae*, *D. biconispora*, *D. limonicola*, *D. masirevicii*, *D. passifloricola, D. perseae*, and *D. sennae* on *C. grandis*. This study provides the first intensive study of endophytic *Diaporthe* species on *C. grandis* cv. tomentosa in China. These results will improve the current knowledge of *Diaporthe* species associated with *C. grandis*. The results obtained in this study will also help to understand the potential pathogens and biocontrol agents and to develop a platform in disease management.

## Introduction

*Diaporthe* Nitschke (1870) belongs to *Diaporthaceae* (Senanayake et al., 2017) which comprises plant pathogens, endophytes, and saprobes (Udayanga et al., 2011; Huang et al., 2013, 2015). In earlier studies, the classification of *Diaporthe* depended mainly on morphology, culture characteristics, and host affiliations (Udayanga et al., 2011). However, with the development of molecular methods, species identification based on multigene phylogeny together with morphological characters provided a more accurate identification (Udayanga et al., 2011; Gao et al., 2017). In recent studies, ITS, *tef1*, *tub2*, *cal*, and partial histone (*HIS*) genes have been used for species delimitation in this genus (Udayanga et al., 2014a; Santos et al., 2017; Guarnaccia and Crous, 2018; Manawasinghe et al., 2019). *Diaporthe* species have a worldwide distribution and diverse host associations (Udayanga et al., 2014b). More than one *Diaporthe* species have been reported on a single host (Guarnaccia and Crous, 2018; Manawasinghe et al., 2019) and one species can be associated with more than one host in the same country or region (Santos and Phillips, 2009; Diogo et al., 2010; Guarnaccia et al., 2018b; Manawasinghe et al., 2019).

*Diaporthe* species are well-known pathogens on economically important crops and woody hosts (Udayanga et al., 2011) and many of these diseases were previously known as “Phomopsis diseases.” Rossman et al. (2014) proposed that the genus name *Diaporthe* should take priority over *Phomopsis*. *Diaporthe* species are widely associated with blights, cankers, diebacks, melanose, and stem-end rots (Udayanga et al., 2014b; Guarnaccia and Crous, 2017; Manawasinghe et al., 2019). There are 19 *Diaporthe* species associated with soybean (*Glycine max* L.) and several of them cause leaf and pod blights and seed decay (Udayanga et al., 2014b; Farr and Rossman, 2020). *Diaporthe* species have been recorded causing leaf spots on *Camellia* spp. (Gao et al., 2016, 2017; Guarnaccia and Crous, 2018). On *Citrus* spp. these taxa have been reported to cause melanosis, stem-end rot, and gummosis (Mondal et al., 2007; Udayanga et al., 2014a; Guarnaccia and Crous, 2017, 2018). The causal organism of sunflower stem canker *D. helianthi* can cause yield loss of up to 40% in Europe and United States (Thompson et al., 2011). *Diaporthe* species are associated with Phomopsis cane and leaf spot on grapevine worldwide (Guarnaccia and Crous, 2018; Manawasinghe et al., 2019). Worldwide, 27 *Diaporthe* species have been reported on grapevines and each species and species combination develop different symptoms (Manawasinghe et al., 2019). Moreover, *Diaporthe* also associated as endophytes on grapevines (Guarnaccia and Crous, 2018).

In China, *Diaporthe* species have been reported as pathogens causing blights, dieback, scab, stem-end rots and trunk diseases in different hosts such as, *Camellia* (Gao et al., 2017), *Citrus* (Huang et al., 2013), *Coffea* (Gao et al., 2017), *Elaeagnus* (Gao et al., 2017), *Lithocarpus* (Gao et al., 2017), *Senna* (Yang et al., 2017a), and *Vitis* (Dissanayake et al., 2015; Manawasinghe et al., 2019). Furthermore, *Diaporthe* species have been reported as saprobes on dead plants and as freshwater fungi (Hu et al., 2012; Huang et al., 2013). Huang et al. (2015) identified 16 *Diaporthe* species including seven new *Diaporthe* species from *Citrus*, adding evidence to the endophytic species richness of *Diaporthe* in *Citrus* plants. Although there are several studies on *Diaporthe* associated with *Citrus* (Huang et al., 2013; Guarnaccia and Crous, 2017), the endophytic fungi associated with *C. grandis* and their relationships with the plant are unknown.

*Diaporthe* species are often reported as endophytes (Murali et al., 2006; Botella and Diez, 2011; Huang et al., 2015) and they may provide several advantages to the plants. They possibly contribute to resistance against pathogens and might also act as a secondary defence layer of the associated plant (Hyde and Soytong, 2008; Dini-Andreote, 2020). It is important to explore the relationship between natural products from the plant and its endophytes (Alvin et al., 2014) since this might reveal novel compounds with antimicrobial activities and medicinal properties. The objectives of the present study were to isolate and identify endophytic *Diaporthe* species associated with healthy *C. grandis* cv. “Tomentosa” trees collected in Huazhou, Guangdong, China. Detailed descriptions of novel species identified based on molecular phylogeny and morphology are provided.

## Materials and Methods

### Sampling and Isolation of Endophytic Fungi

Healthy *C. grandis* cv. “Tomentosa” leaves, twigs, and fruits were collected randomly from a *Citrus* orchard in Huazhou city, Guangdong Province, China in May 2019. Samples were placed in plastic zip-lock bags containing sterilized wet cotton to prevent drying, taken to the laboratory and isolations were made on the same day. The samples were initially washed with tap water and then with sterile water. The leaves were then cut into 3 mm × 3 mm segments, twigs into pieces 3 mm long, and the fruits into 3 cm^3^ cubes. Each piece was surface sterilized by dipping sequentially into 75% ethanol for 40 s, 2.5% NaOCl (sodium hypochlorite) for 90 s, rinsed with three changes of sterile water, dried on sterilized filter paper and then placed on potato dextrose agar (PDA). Plates were incubated at 25°C with 12 h dark and 12 h fluorescent light. In total, 80 tissue segments were obtained from leaves, twigs, and fruits. Fungi growing from the edges of the tissue were sub-cultured on fresh PDA plates. To obtain pure cultures, single spore isolation was carried out (Chomnunti et al., 2011).

### DNA Extraction and PCR Amplification

Mycelia were scraped from 7 days old pure cultures growing on PDA and total genomic DNA was extracted using the CTAB method (Sun et al., 2009). The ITS region was amplified and sequenced with primers ITS1/ITS4 (White et al., 1990). BLAST searches in GenBank with the ITS sequences provided genus level identifications. Once the BLAST results confirmed the isolates as *Diaporthe* species, additional three gene regions, namely translation elongation factor-1α (*tef1*), β-tubulin (*tub2*), and calmodulin (*cal*) were amplified and sequenced. The protocols for PCR amplification were followed as given in Udayanga et al. (2012) and Manawasinghe et al. (2019). The primer pairs and their respective amplification conditions are given in Table 1. Positive PCR amplicons were observed on 1% agarose electrophoresis gel. Sequencing (forward direction, both directions when necessary) was done by Tianyi Huiyuan Biotechnology Co., Ltd., Guangzhou, China. Initial sequence quality was checked with BioEdit 7.25 (Hall, 2006). All sequence data generated in this study were submitted to GenBank (Supplementary Table 1).

**TABLE 1:** Gene regions and respective primer pairsused in the study.

Gene region	Primers	Sequence5′-3′	Optimized PCR protocols	References
ITS	ITS1	TCCGTAGGTGAACCTGCGG	94°C: 5 min, (94°C: 30 s, 55°C: 50 s, 72°C: 1 min) × 34 cycles 72°C: 10 min	White et al., 1990
	ITS4	TCCTCCGCTTATTGATATGC		
*tub2*	BT2a	GGTAACCAAATCGGTGCTGCTTTC	94°C: 5 min, (94°C: 30 s, 58°C: 50 s, 72°C: 1 min) × 34 cycles 72°C: 10 min	Glass and Donaldson, 1995
	Bt2b	ACCCTCAGTGTAGTGACCCTTGGC		
*tef1*	EF1-728F	CATCGAGAAGTTCGAGAAGG	95°C: 5 min, (95°C: 30 s, 58°C: 30 s, 72°C: 1 min) × 34 cycles 72°C: 10 min	Carbone and Kohn, 1999
	EF1-986R	TACTTGAAGGAACCCTTACC		
*cal*	CAL-228F	GAGTTCAAGGAGGCCTTCTCCC	95°C: 5 min, (95°C: 30 s, 55°C: 50 s, 72°C: 1 min) × 34 cycles 72°C: 10 min	Carbone and Kohn, 1999
	CAL-737R	CATCTTCTGGCCATCATGG		

### Phylogenetic Analysis

For the phylogenetic analysis, sequences of reference *Diaporthe* species and related taxa were obtained from NCBI GenBank and selected by reference to relevant published trees (Guarnaccia et al., 2018b; Yang et al., 2018a; Hyde et al., 2019; Long et al., 2019; Manawasinghe et al., 2019; Supplementary Table 1). Sequences for each locus were aligned together with the sequences obtained in the present study using MAFFT (Katoh and Toh, 2010)^1^. Alignments were checked and manually adjusted where necessary with BioEdit v. 5 (Hall, 2006). Phylogenetic analyses were conducted by maximum likelihood (ML) in RAxML (Silvestro and Michalak, 2010), maximum parsimony (MP) in PAUP (v4.0) (Swofford, 2003) and Bayesian analyses (BI) in MrBayes (v. 3.0b4) (Ronquist and Huelsenbeck, 2003). The final analysis was made with the concatenated data set of ITS, *tef1*, *tub2*, *cal* following Dissanayake et al. (2017a); Guarnaccia et al. (2018b) and Manawasinghe et al. (2019).

For the MP analysis, ambiguous regions in the alignment were excluded and gaps were treated as missing data. Tree stability was evaluated with 1,000 bootstrap replications. Zero-length branches were collapsed, and all parsimonious trees were saved. Tree parameters; tree-length (TL), consistency index (CI), retention index (RI), relative consistency index (RC), and homoplasy index (HI) were calculated. Kishino-Hasegawa tests (KHT) were conducted to evaluate differences between the trees inferred under different optimality criteria (Kishino and Hasegawa, 1989). MrModeltest v. 2.3 (Nylander, 2004) was used to determine the evolutionary models for each locus to be used in Bayesian and maximum likelihood analyses. The maximum likelihood analyses were conducted using RAxML-HPC2 on XSEDE (8.2.8) (Stamatakis, 2014) in the CIPRES Science Gateway platform (Miller et al., 2010). The GTR + I + G evolutionary model was employed with 1,000 non-parametric bootstrapping iterations. Bayesian analysis was performed in MrBayes v. 3.0b4 (Ronquist and Huelsenbeck, 2003). Six simultaneous markov chains were run for 10^6^ generations, sampling the trees at every 200th generation. From the 5,000 trees obtained, the first 2,000 representing the burn-in phase were discarded. The remaining 3,000 trees were used to calculate posterior probabilities (BYPPs) in a majority rule consensus tree. The final sequence alignment generated in this study was submitted to TreeBASE ID 26384^2^. Taxonomic novelties were submitted to Index Fungorum^3^ and Faces of Fungi database (Jayasiri et al., 2015). Newly generated sequences were deposited in GenBank.

### Morphological Characterization

Agar plugs (5mm diam) were taken from actively growing cultures on PDA and transferred onto PDA, malt extract agar (MEA) (Crous et al., 2009) and pine needle agar (PNA: 2% WA with three sterilized pine needles) (Smith et al., 1996) plates and incubated at 25°C with 12 hours of alternating darkness and fluorescent light per day for over a month to induce sporulation (Gomes et al., 2013; Huang et al., 2015). Colony characters and pigmentation on MEA and PDA were recorded after 7, 15, and 30 days. Colony color (upper and reverse) was described by referring to the color charts of Rayner (1970). Colony diameters were measured after 3–7 days. Digital images of morphological structures (shape, size, and color) were recorded with an Eclipse 80i photographic microscope (Nikon, Japan). Conidial length and width were measured for 40 conidia per isolate using NIS-Elements BR 3.2, and the mean values were calculated with their standard deviations (SDs).

## Results

**FIGURE 1:**
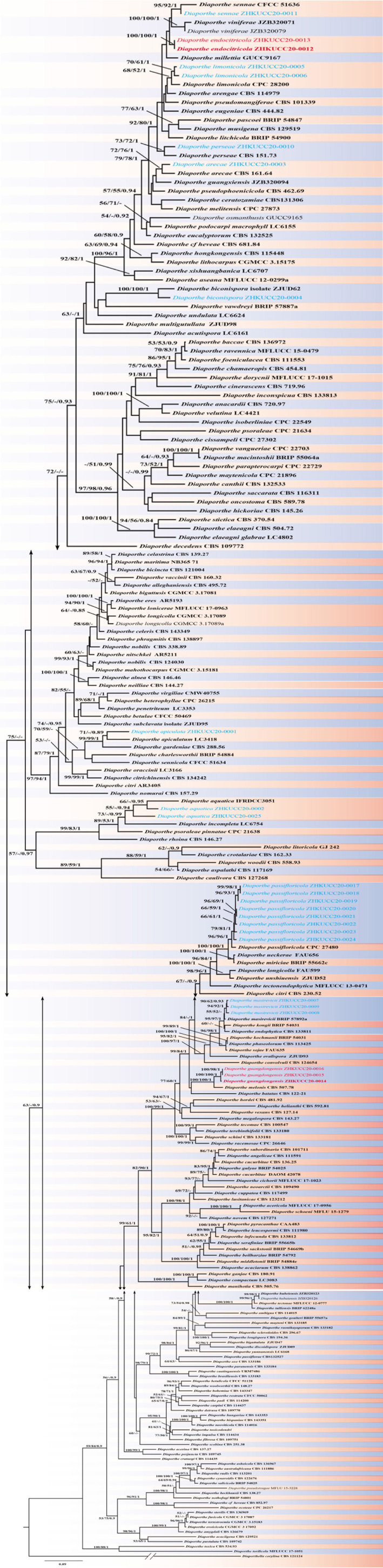
The best scoring RAxML tree obtained using the combined dataset of ITS, *tef1*, *tub2*, and *cal* sequences. *Diaporthella corylina* (CBS 121124) was used to root the tree. Bootstrap support values equal to or greater than 50% in ML and MP and BYPP equal or greater than 0.95 are shown as ML/MP/BYPP above the respective node. The isolates belonging to the current study are given in blue for known species, and novel taxa are shown in red. Ex-type strains are bold. Expected number of nucleotide substitutions per site is represented by the scale bar.

In total 24 endophytic *Diaporthe* strains were obtained (two from leaves, five from twigs and 17 from fruits). The phylogenetic analysis of a combined ITS, *tef1*, *tub2*, and *cal* sequence alignment was conducted using 222 *Diaporthe* strains (including type strains). The phylogenetic tree was rooted with *Diaporthella corylina* (CBS 121124). The final ML tree topology was similar to the MP and BI trees. The best scoring ML tree with a final likelihood value of −55258.653244 is given in Figure 1. The matrix consisted of 1,567 distinct alignment patterns, with 32.05% of undetermined characters or gaps. Estimated base frequencies were as follows: A = 0.215752, C = 0.315593, G = 0.242350, T = 0.226306; substitution rates AC = 1.197240, AG = 3.454524, AT = 1.225071, CG = 0.944927, CT = 4.809973, GT = 1.000000; gamma distribution shape parameter α = 0.511913. The dataset consisted of 2,134 characters with 779 constant characters and 1,072 parsimony-informative and 283 parsimony-uninformative characters. The maximum number of trees generated was 5,000, and the most parsimonious trees had a length of 10930 steps (CI = 0.252, RI = 0.731, RC = 0.184, HI = 0.748). In the ML tree, isolates from this study clustered together with nine known *Diaporthe* species and as two novel phylogenetic lineages. Species descriptions and illustrations of all these species are given below.

### Taxonomy

#### Diaporthe apiculata

**FIGURE 2:**
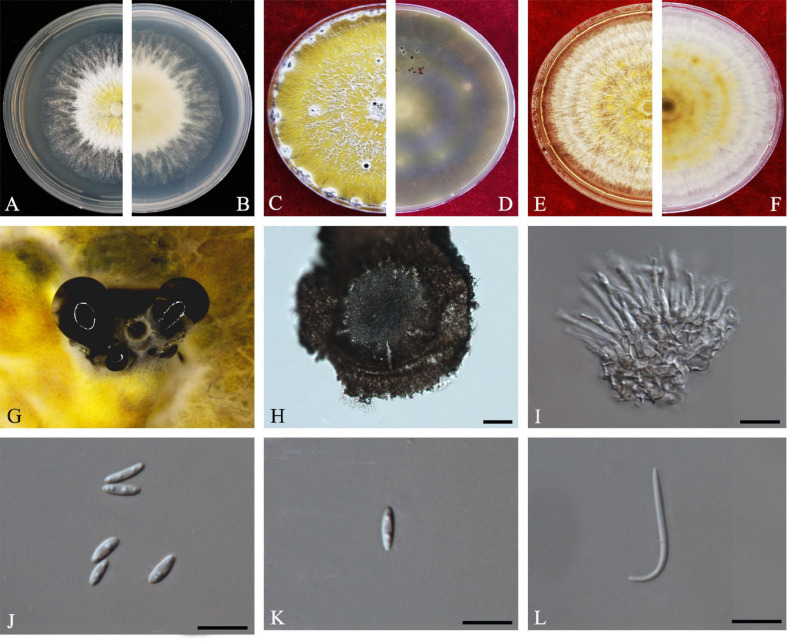
*Diaporthe apiculata* (ZHKUCC 20-0001). **(A,B)** Colonies on PDA after 4 days at 25°C, **(C,D)** Colonies on PDA after 30 days at 25°C, **(E,F)** Colonies on MEA after 14 days at 25°C, **(G)** Conidiomata sporulating on PNA, **(H)** Pycnidium, **(I)** Conidiogenous cells, **(J,K)** Alpha conidia, and **(L)** Beta conidia. Scale bars: **(H)** = 100 μm; **(I–L)** = 10 μm.

Y.H. Gao and L. Cai, *Syst Biodivers* 14: 106 (2016) *Index Fungorum number*: 811217, *Faces of fungi Number*: FoF 08403 (Figure 2).

*Endophytic* on *C. grandis* “Tomentosa” twigs. **Sexual morph:** not observed. **Asexual morph:** *Pycnidia* 140–490 μm × 130–450 μm ($\bar{x}$ = 377 μm × 303 μm), hemispherical, erumpent, coated with white aerial mycelium. *Conidiophores* 10–22 μm × 1–2 μm ($\bar{x}$ = 15 ± 3.5 μm × 2 ± 0.3 μm), subcylindrical, unbranched, densely aggregated, straight to sinuous. *Alpha conidia* 6–8 μm × 2–3 μm ($\bar{x}$ = 7 ± 0.7 μm × 3 ± 0.3 μm), cylindrical to ellipsoid, hyaline, aseptate, biguttulate or three to four guttules. *Beta conidia* 19–31 μm × 1–2 μm ($\bar{x}$ = 26 ± 3 μm × 2 ± 0.2 μm), filiform, hyaline, and tapering toward both ends, hamate or curved.

*Culture Characteristics*: Colonies on PDA reach 75 mm diam. after 5 days at 25°C. White fluffy aerial mycelium, margin filiform. Later turning to brownish yellow, pigmentation developing from the center. Reverse initially white and then turning brownish-yellow from the center, some became brownish-green.

*Material examined*: CHINA, Guangdong Province, Huazhou, isolated from a healthy twig of *C. grandis* “Tomentosa,” May 2019, ZY Dong and YX Shu, (dried culture ZHKU 20-0001); living culture ZHKUCC 20-0001).

*Habitat and host*: *Betula* spp. (Du et al., 2016); *Brassica oleracea* var. *acephala* (Turkkan et al., 2020); *Camellia sinensis* (Gao et al., 2016), *Camptotheca acuminate* (Yang et al., 2017b); *Juglans regia* (Fan et al., 2018); peach (Dissanayake et al., 2017a); *Sambucus williamsii* (Caprifoliaceae) (Yang et al., 2018a); *Schisandra chinensis* (Schisandraceae) (Yang et al., 2018a); *Senna bicapsularis* (Yang et al., 2017a).

*Known distribution*: China; Turkey (Farr and Rossman, 2020).

*Note*: A single isolate in the present study clustered together with the *Diaporthe apiculata* ex-type strain (CGMCC 3.17533) with 71% maximum likelihood bootstrap value and 0.89 Bayesian posterior probabilities. Colony morphology and conidial dimensions of the present isolate (ZHKUCC 20-0001) were similar to those in the original description of *D. apiculata* (Gao et al., 2016). *Diaporthe apiculata* was introduced by Gao et al. (2016) for a species associated with healthy leaves of *Camellia*. This is the first report of *D. apiculata* on *C. grandis* cv. “Tomentosa” (Farr and Rossman, 2020).

#### Diaporthe aquatica

**FIGURE 3:**
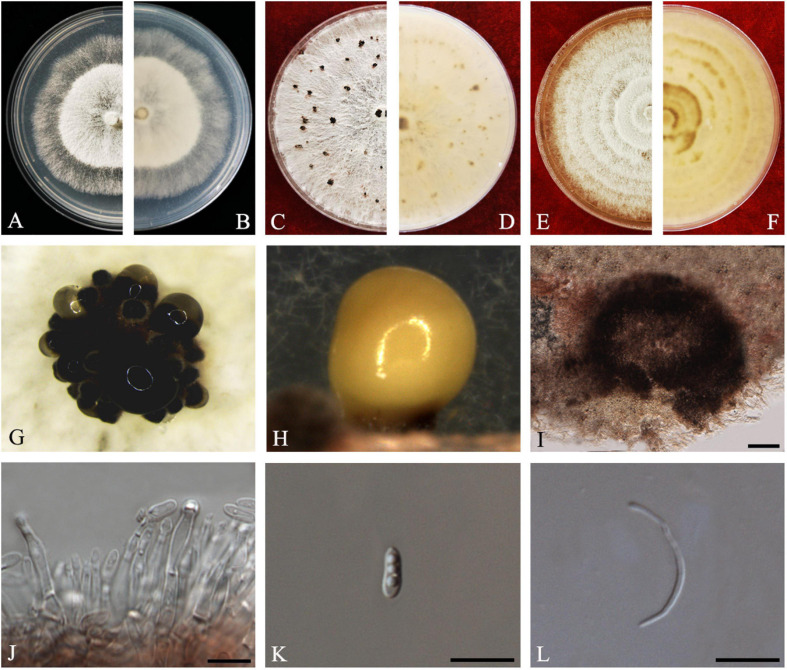
*Diaporthe aquatica* (ZHKUCC 20-0002). **(A,B)** Colonies on PDA after 4 days at 25°C, **(C,D)** Colonies on PDA after 30 days at 25°C, **(E,F)** Colonies on MEA after 14 days at 25°C, **(G,H)** Conidiomata sporulating on PDA, **(I)** Pycnidium, **(J)** Conidiogenous cells, **(K)** Alpha conidia, and **(L)** Beta conidia. Scale bars: **(I)** = 100 μm; **(J–L)** = 10 μm.

D.M. Hu, L. Cai and K.D. Hyde, *Mycologia* 104(6): 1481 (2012) *Index Fungorum number*: IF564857, *Faces of fungi Number*: FoF08404 (Figure 3).

*Endophytic* on *C. grandis* “Tomentosa” fruits. **Sexual morph:** not observed. **Asexual morph:** *Pycnidia* 150–510 μm × 100–500 μm ($\bar{x}$ = 294 ± 102 μm × 239 ± 98 μm), globose to subglobose, black, coriaceous, immersed to semi-immersed, single to clustered, conidia produced in yellow to brown creamy drops. *Conidiophores* 10–60 μm × 2–3 μm ($\bar{x}$ = 22 ± 9 μm × 2 ± 0.4 μm), cylindrical, hyaline. *Alpha conidia* 6–8 μm × 2–3 μm ($\bar{x}$ = 7 ± 0.6 μm × 3 ± 0.4 μm), ellipsoidal to fusiform, hyaline, slightly constricted in the middle, some with one end rounded and the others acute, with two to five guttules. *Beta conidia* 6–43 μm × 1–2 μm ($\bar{x}$ = 25 ± 9 μm × 1 ± 0.3 μm), filiform, hyaline, aseptate, and curved at one end.

*Culture Characteristics*: Colonies on PDA reach 85 mm diam. after 5 days at 25°C. Aerial mycelium, white, undulate margin, forming concentric rings of pycnidia. Reverse white, and then turning to brown-yellow from the center.

*Material examined*: CHINA, Guangdong Province, Huazhou, isolated from healthy fruits of *C. grandis* “Tomentosa,” May 2019, ZY Dong and YX Shu, (dried culture ZHKU 20-0002); living culture ZHKUCC 20-0002).

*Habitat and host*: Freshwater fungus (Hu et al., 2012).

*Known distribution*: China (Hu et al., 2012).

*Note*: Isolate ZHKUCC 20-0002 obtained in this study clusters together with the ex-type isolate of *Diaporthe aquatica* (IFRDCC3051) with 66% maximum likelihood bootstrap and 0.95 Bayesian posterior probability values. Alpha and beta conidia of this strain have a similar length to *Diaporthe aquatica* (Hu et al., 2012). So far, this species has been reported only as a freshwater fungus from China (Hu et al., 2012). This is the first report of *D. aquatica* on *C. grandis* cv. “Tomentosa” (Farr and Rossman, 2020).

#### Diaporthe arecae

(H.C. Srivast., Zakia and Govindar) R.R. Gomes, C. Glienke and Crous, *Persoonia* 31: 16 (2013).

**FIGURE 4:**
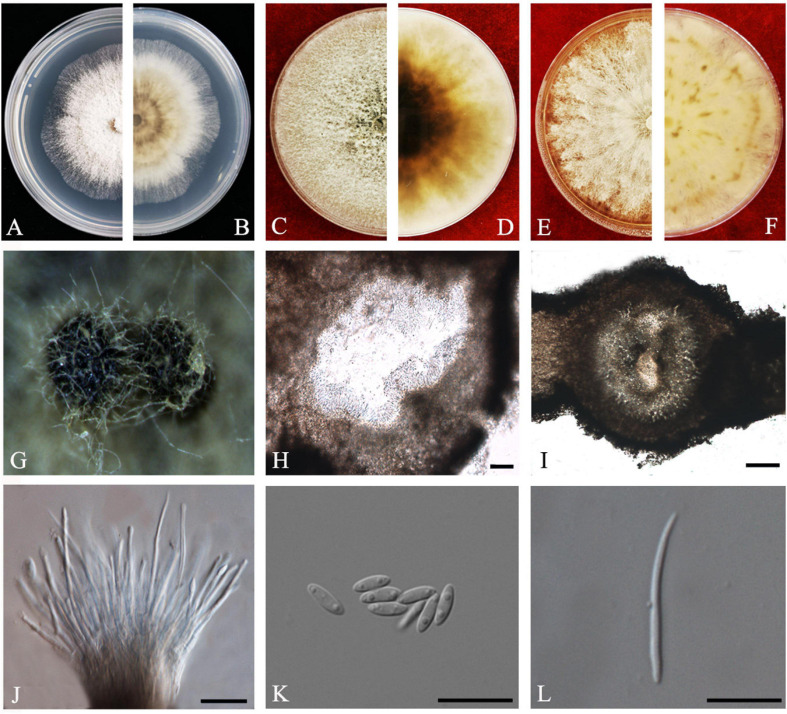
*Diaporthe arecae* (ZHKUCC 20-0003). **(A,B)** Colonies on PDA after 4 days at 25°C, **(C,D)** Colonies on PDA after 30 days at 25°C, **(E,F)** Colonies on MEA after 14 days at 25°C, **(G)** Conidiomata sporulating on PNA, **(H,I)** Pycnidium, **(J)** Conidiogenous cells, **(K)** Alpha conidia, and **(L)** Beta conidia. Scale bars: **(H,I)** = 100 μm; **(J–L)** = 10 μm.

*Index Fungorum number*: 802924, *Faces of fungi*: FoF 08405 (Figure 4).

*Endophytic* on *C. grandis* “Tomentosa” twigs. **Sexual morph:** not observed. **Asexual morph:** *Pycnidia* 95–730 μm × 87–640 μm ($\bar{x}$ = 419 ± 167 μm × 361 ± 164 μm), subglobose or lageniform. *Conidiophores* 7–38 μm × 1–3 μm ($\bar{x}$ = 22 ± 8 μm × 2 ± 0.4 μm), cylindrical, hyaline. *Alpha conidia* 6–10 μm × 2–3 μm ($\bar{x}$ = 8 ± 0.8 μm × 2 ± 0.4 μm), cylindrical to ellipsoid, hyaline, aseptate, both ends acute, mostly biguttulate. *Beta conidia* 17–26 μm × 1–2 μm ($\bar{x}$ = 22 ± 2 μm × 2 ± 0.2 μm), filiform, hyaline, aseptate, curved at one end, one end blunt and the other end pointed.

*Culture Characteristics*: After 5 days at 25°C colonies reach 85 mm diam. on PDA. White radial, margin undulate. Reverse white, becoming tawny then dark brown from the center.

*Material examined*: CHINA, Guangdong Province, Huazhou, isolated from a healthy twig of *C. grandis* “Tomentosa,” May 2019, ZY Dong and YX Shu, (dried cultures ZHKU 20-0003); living cultures ZHKUCC 20-0003).

*Habitat and host*: *Areca catechu* (Gomes et al., 2013), *Citrus grandis*, *Citrus limon*, *Citrus reticulata*, *Citrus unshiu* (Huang et al., 2015), *Citrus sinensis* (Huang et al., 2015; Guarnaccia and Crous, 2017), *Citrus* sp. (Gomes et al., 2013; Guarnaccia and Crous, 2017). *Mangifera indica* (Lim et al., 2019).

*Known distribution*: India, China, Suriname, Malaysia (Farr and Rossman, 2020).

*Note*: The single isolate (ZHKUCC 20-0003) obtained in this study clustered with *Diaporthe arecae* (CBS 161.64) with 72% maximum likelihood, 76% maximum parsimony bootstrap values and 1.0 Bayesian posterior probability values. The isolate in this study was morphologically similar to the *Diaporthe arecae* type description (Gomes et al., 2013) by developing conidia with similar shapes and dimensions. This species was first reported on *Citrus* in China by Huang et al. (2015). *Diaporthe arecae* has been reported as being saprobic and pathogenic on *Citrus* and the pathogenicity of this species was confirmed by Guarnaccia and Crous (2017).

#### Diaporthe biconispora

**FIGURE 5:**
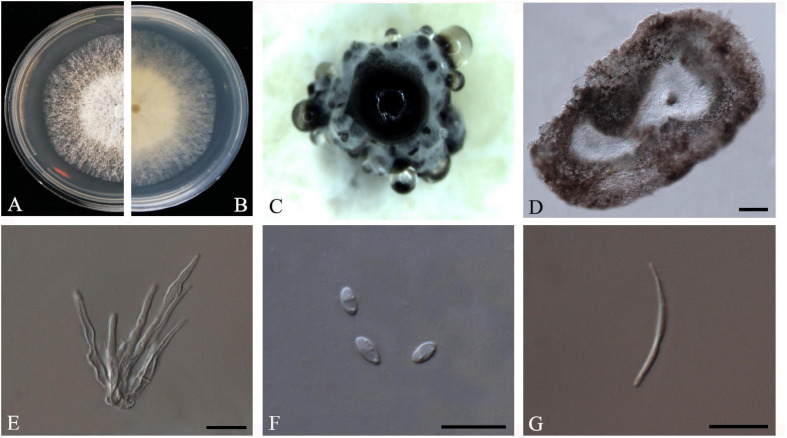
*Diaporthe biconispora* (ZHKUCC 20-0004). **(A,B)** Colonies on PDA after 4 days at 25°C, **(C)** Conidiomata sporulating on PDA, **(D)** Pycnidium, **(E)** Conidiogenous cells, **(F)** Alpha conidia, and **(G)** Beta conidia. Scale bars: **(D)** = 100 μm; **(E–G)** = 10 μm.

F. Huang, K.D. Hyde and H.Y. Li, *Fungal Biology* 119(5): 338 (2015) *Index Fungorum number*: IF810578, *Faces of fungi*: FoF08406 (Figure 5).

*Endophytic* on *C. grandis* “Tomentosa” leaves. **Sexual morph:** not observed. **Asexual morph:** *Pycnidia* 150–770 μm × 75–430 μm ($\bar{x}$ = 275 μm × 210 μm), globose to sub-globose, brown to dark brown, conidia produced in white to transparent creamy and yellow drops. *Conidiophores* 10–45 μm × 1–4 μm ($\bar{x}$ = 22 ± 9 μm × 2 ± 0.6 μm), cylindrical to sub-cylindrical, simple, hyaline. *Alpha conidia* 4–7 μm × 2–3 μm ($\bar{x}$ = 6 ± 1 μm × 3 ± 0.3 μm), cylindrical to ellipsoid, hyaline, aseptate, biguttulate or multiguttulate. *Beta conidia* 24–37 μm × 1–2 μm ($\bar{x}$ = 30 ± 4 μm × 2 ± 0.2 μm), filiform, hyaline, aseptate and curved at one end.

*Culture Characteristics*: Cultures reach 75 mm diam. on PDA at 25°C after 5 days. White to light yellow, aerial mycelium, circular, entire margin. Reverse light yellow, black pigmentation at the center.

*Material examined*: CHINA, Guangdong Province, Huazhou, isolated from a healthy leave of *C. grandis* cv. “Tomentosa,” May 2019, ZY Dong and YX Shu, (dried culture ZHKU 20-0004); living culture ZHKUCC 20-0004).

*Habitat and host*: *Betula* spp. (Du et al., 2016) *Citrus grandis* (Huang et al., 2015), *Citrus maxima* (Fan et al., 2018), *Citrus sinensis* (Huang et al., 2015), *Fortunella margarita* (Huang et al., 2015), *Senna bicapsularis* (Yang et al., 2017a).

*Known distribution*: China (Farr and Rossman, 2020).

*Note:* One isolate in this study clustered with *Diaporthe biconispora* (ZJUD62) with 100% maximum likelihood, 100% maximum parsimony bootstrap values and 1.0 Bayesian posterior probability values. Colony morphology and conidial characters of this isolate (ZHKUCC 20-0004) are similar to those in the type description of *D. biconispora* (Huang et al., 2015). The isolate (ZHKUCC 20-0004) obtained in this study developed beta conidia whereas type strain did not (Huang et al., 2015). *Diaporthe biconispora* was introduced by Huang et al. (2015) as an endophytic fungus from *Citrus grandis, C. sinensis*, and *Fortunella margarita*.

#### Diaporthe endocitricola

**FIGURE 6:**
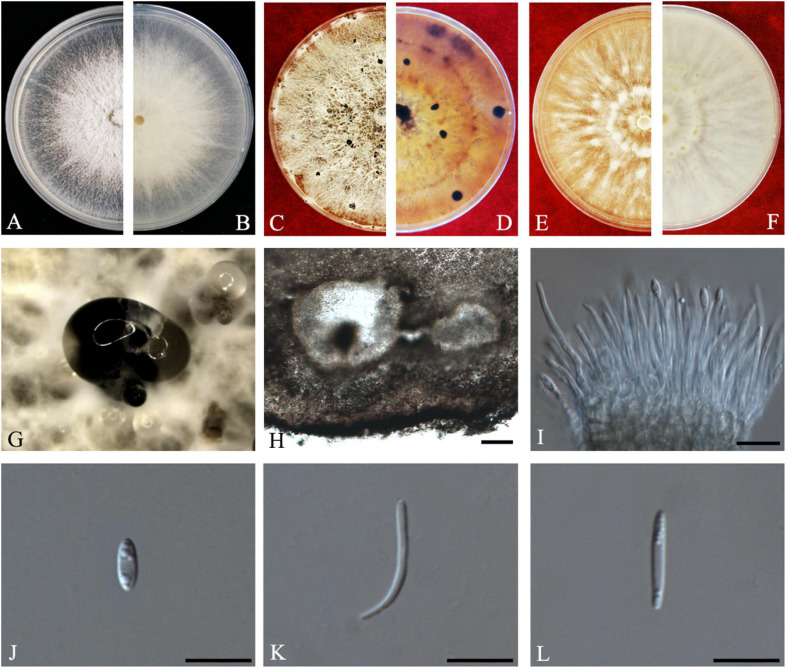
*Diaporthe endocitricola* (ZHKUCC 20-0012, Holotype). **(A,B)** Colonies on PDA after 4 days at 25°C, **(C,D)** Colonies on PDA after 30 days at 25°C, **(E,F)** Colonies on MEA after 14 days 25°C, **(G)** Conidiomata sporulating on PDA, **(H)** Pycnidium, **(I)** Conidiogenous cells, **(J)** Alpha conidia, **(K)** Beta conidia, and **(L)** Gamma conidia. Scale bars: **(H)** = 100 μm; **(I–L)** = 10 μm

Z.Y. Dong, M. Luo, M.M. Xiang, K.D. Hyde**, sp. *nov*.** *Index fungorum number*: IF557628, *Faces of fungi:* FoF 08409 (Figure 6).

*Etymology*–In reference to its endophytic nature in *Citrus grandis*.

*Holotype*–ZHKUCC 20-0012.

*Endophytic* on *C. grandis* “Tomentosa” fruits. **Sexual morph:** not observed. **Asexual morph:** *Pycnidia* 124–790 μm × 111–635 μm ($\bar{x}$ = 353 ± 170 μm × 289 ± 134 μm), multilocular, subglobose or lageniform, ostiolate, produced in hyaline to dark black creamy drops. *Conidiophores* 12–40 μm × 1–3 μm ($\bar{x}$ = 26 ± 7 μm × 2 ± 0.3 μm), formed from the inner layer of the locular wall, cylindrical, hyaline. *Alpha conidia* 6–8 μm × 2–3 μm ($\bar{x}$ = 7 ± 1 μm × 3 ± 0.3 μm), cylindrical to ellipsoid, hyaline, aseptate, mostly multiguttulate with guttules focus on both ends. *Beta conidia* 12–30 μm × 1–2 μm ($\bar{x}$ = 19 ± 4 μm × 2 ± 0.2 μm), filiform, hyaline, aseptate, straight or curved at one end. *Gamma conidia* fusiform, hyaline, multiguttulate.

*Culture characteristics*: Cultures reach 85 mm diam., after 5 days on PDA at 25°C. White with radial hyphal growth at the rim, circular form with entire margin, with some irregular conidiomata after 20 days. Reverse white becoming yellow-brown, with zonations.

*Material examined*: CHINA, Guangdong Province, Huazhou, isolated from healthy fruits of *C. grandis* cv. “Tomentosa,” May 2019, ZY Dong and YX Shu, (dried culture ZHKU 20-0012 holotype and ZHKU 20-0013 paratype); living cultures ZHKUCC 20-0012 ex-type and ZHKUCC 20-0013 ex-paratype.

*Habitat and host*: On healthy fruits of *C. grandis* cv. “Tomentosa”

*Known distribution*: China (Huazhou, Guangdong provinces).

*Note:* In the multigene phylogenetic tree developed using ITS, *tef1*, *tub2*, and *cal*, two isolates (ZHKUCC 20-0012 and ZHKUCC 20-0013) from the present study developed a sister clade to *D. millettiae* (GUCC9167) with 100% maximum likelihood, 100% maximum parsimony bootstrap and 1.0 Bayesian posterior probability values. Morphologically, *Diaporthe endocitricola* has more guttules in alpha conidia (mostly multiguttulate with guttules focused on both ends) than *D. millettiae* (mostly biguttulate) (Long et al., 2019). In addition to that, *D. endocitricola* has longer conidiophores (12–38 μm × 1–3 μm) than *D. millettiae* (10–23 × 1–2.5) (Long et al., 2019). *Diaporthe endocitricola* develops gamma conidia while *D. millettiae* does not (Long et al., 2019). When of four gene regions compared between *D. endocitricola* and *D. millettiae*, *D. millettiae* has 1.6% nucleotide differences in ITS along 549 nucleotides. In comparisons of protein-coding regions, 3% differences in *tef1* (332 nucleotides), 4% differences in *tub2* (513 nucleotides), 1% differences in *cal* (440 nucleotides). Considering both morphological and molecular data, these isolates were identified as a novel species.

#### Diaporthe guangdongensis

**FIGURE 7:**
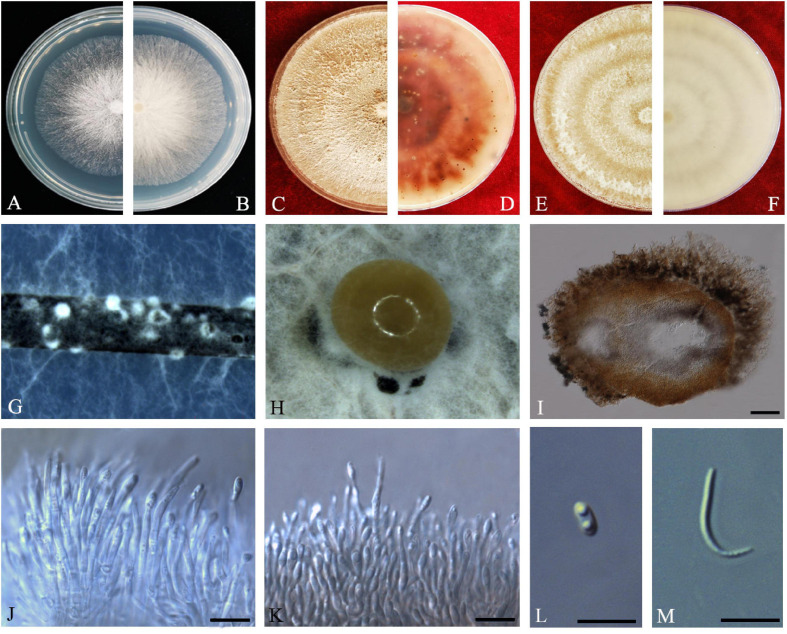
*Diaporthe guangdongensis* (ZHKUCC 20-0014 Holotype). **(A,B)** Colonies on PDA after 2 days at 25°C, **(C,D)** Colonies on PDA after 30 days at 25°C, **(E,F)** Colonies on MEA after 14 days at 25°C, **(G)** Conidiomata sporulating on PNA, **(H)** Conidiomata sporulating on PDA, **(I)** Pycnidium, **(J,K)** Conidiogenous cells, **(L)** Alpha conidia, and **(M)** Beta conidia. Scale bars: **(H)** = 100 μm; **(I–M)** = 10 μm.

Z.Y. Dong, M. Luo, M.M. Xiang, K.D. Hyde, **sp. *nov*.** *Index Fungorum number*: IF557627, *Faces of fungi*: FoF08410 (Figure 7).

*Etymology*–In reference to the Guangdong province, from where the samples were collected.

*Holotype*–ZHKUCC 20-0014.

*Endophytic* on *C. grandis* “Tomentosa” fruits. **Sexual morph:** not observed. **Asexual morph:** *Pycnidia* 130–515 μm × 100–390 μm ($\bar{x}$ = 286 ± 101.4 μm × 229 ± 76 μm), subglobose or lageniform, solitary, with neck, conidia produced in yellowish-brown to blackish green creamy drops. *Conidiophores* 11–46 μm × 1–3 μm ($\bar{x}$ = 24 ± 9 μm × 2 ± 0.4 μm), branched, densely aggregated, cylindrical, straight to sinuous, hyaline, 1–3-septate. *Alpha conidia* 6–8 μm × 2–4 μm ($\bar{x}$ = 7 ± 0.5 μm × 3 ± 0.4 μm), hyaline, unicellular, fusiform to ellipsoidal, with two big guttules. *Beta conidia* 14–35 μm × 1–2 μm ($\bar{x}$ = 21 ± 4 μm × 2 ± 0.2 μm), filiform, hyaline, unicellular, aseptate and curved at one end.

*Culture Characteristics*: Cultures reach 85 mm diam. after 3 days on PDA at 25°C. White turn to yellowish-white with time, circular, entire margin. Reverse white and turn to reddish-brown from the center with time.

*Material examined*: CHINA, Guangdong Province, Huazhou, isolated from healthy fruits of *C. grandis* cv. “Tomentosa,” May 2019, ZY Dong and YX Shu, (dried cultures ZHKU 20-0014, holotype and ZHKU 20-0015–16, isotype); living cultures ZHKUCC 20-0014 ex-type and ZHKUCC 20-0015–16 ex-isotype.

*Habitat and host*: On healthy fruit of *C. grandis* cv. “Tomentosa”

*Known distribution*: China (Huazhou, Guangdong provinces).

*Note:* In the phylogenetic tree developed using ITS, *tef1*, *tub2*, and *cal*, three isolates obtained in this study developed a sister clade to *D. melonis* (CBS 507.78) with 100% maximum likelihood, 100% maximum parsimony bootstrap, and 1.0 Bayesian posterior probability values. Morphologically, *Diaporthe guangdongensis* has shorter alpha conidia (6–8 μm × 2–4 μm, $\bar{x}$ = 6.5 ± 0.5 μm × 2.9 ± 0.4 μm) than *D. melonis* (6.3–10.3 μm × 2.16–3 μm, $\bar{x}$ = 8.3 × 2.6) (Beraha and O’Brien, 1979). *Diaporthe guangdongensis* has shorter beta conidia (14–35 × 1–2, $\bar{x}$ = 21 ± 4 μm × 2 ± 0.2 μm) than *D. melonis* (8.6–27.7 μm × 1–2 μm, $\bar{x}$ = 24.7 × 1.3 μm). In comparisons of four gene regions between *D. guangdongensis* and *D. melonis*, 1.6% nucleotide difference was observed in ITS along 565 nucleotides. In comparisons of protein-coding regions, 3% differences in *tef1* (352 nucleotides), 1.3% differences in *tub2* (447 nucleotides), 2.8% differences in *cal* (527 nucleotides) were observed. On the basis of these dissimilarities, the three isolates obtained in this study were described as a novel endophytic species associated with *C. grandis* “Tomentosa.”

#### Diaporthe limonicola

**FIGURE 8:**
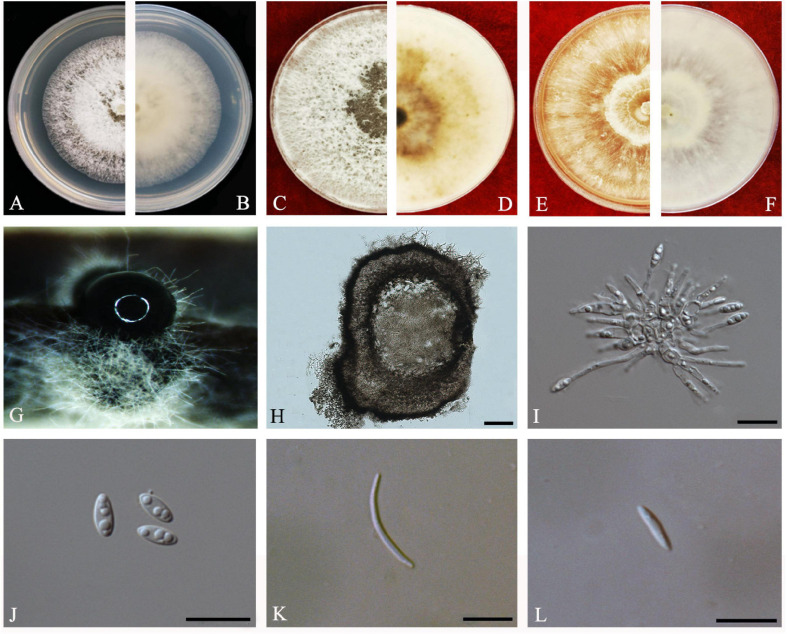
*Diaporthe limonicola* (ZHKUCC 20-0005). **(A,B)** Colonies on PDA after 4 days at 25°C, **(C,D)**. Colonies on PDA after 30 days at 25°C, **(E,F)** Colonies on MEA after 14 days at 25°C, **(G)** Conidiomata sporulating on PNA, **(H)** Pycnidium, **(I)** Conidiogenous cells, **(J)** Alpha conidia, **(K)** Beta conidia, and **(L)** Gamma conidia. Scale bars: **(H)** = 100 μm; **(I–L)** = 10 μm.

Guarnaccia and Crous, IMA Fungus 8(2): 317–334 (2017) *Index Fungorum number*: IF821731, *Faces of fungi*: FoF08407 (Figure 8).

*Endophytic* on *C. grandis* “Tomentosa” leaves. **Sexual morph:** not observed. **Asexual morph:** *Pycnidia* 120–760 μm × 80–660 μm ($\bar{x}$ = 400 μm × 330 μm), subglobose or lageniform, whitish cream to dark brown conidial drops exuded from the ostioles. *Conidiophores* 12–30 μm × 1–2 μm ($\bar{x}$ = 20 ± 7 μm × 2 ± 0.3 μm), cylindrical, hyaline, smooth, densely aggregated. *Alpha conidia* 5–10 μm × 2–3 μm ($\bar{x}$ = 7 ± 1 μm × 2 ± 0.4 μm), cylindrical to ellipsoid, hyaline, aseptate, mono- to biguttulate and acute at both ends. *Beta conidia* 14–29 μm × 1–2 μm ($\bar{x}$ = 22 ± 4 μm × 2 ± 0.3 μm), filiform, hyaline, aseptate, straight or curved, tapering toward both ends. *Gamma conidia* cylindrical, hyaline, multiguttulate.

*Culture Characteristics*: At 25°C colonies reach 85 mm diam. on PDA after 5 days of inoculation. White, circular, entire margin, becoming cream to smoke-gray. Reverse white turning brown and dark gray, dark brown scattered pigmentation. Colonies on MEA at first white, then cream and become yellowish, flat, and dense. Reverse pale brown with conidiomata appearing as brownish dots that become black solitary or aggregated conidiomata at maturity.

*Material examined*: CHINA, Guangdong Province, Huazhou, isolated from a healthy fruit of *C. grandis* cv. “Tomentosa,” May 2019, ZY Dong and YX Shu, (dried cultures ZHKU 20-0005–6); living cultures ZHKUCC 20-0005–6.

*Habitat and host*: *Citrus limon* (Guarnaccia and Crous, 2017).

*Known distribution*: Malta (Guarnaccia and Crous, 2017) and China (this study).

*Note*: Two isolates obtained in this study clustered together with *Diaporthe limonicola* (CBS H-23126) with 68% maximum likelihood, 52% maximum parsimony bootstrap, and 1.0 Bayesian posterior probability values. Morphologically both strains produced similarly shaped and similar sized conidia to the original description of *D. limonicola* (Guarnaccia and Crous, 2017). *Diaporthe limonicola* was introduced by Guarnaccia and Crous (2017) as a species associated with serious trunk and branch cankers of *C. limon*. This is the first report of *D. limonicola* on *C. grandis* cv. “Tomentosa” (Farr and Rossman, 2020).

#### Diaporthe masirevicii

**FIGURE 9:**
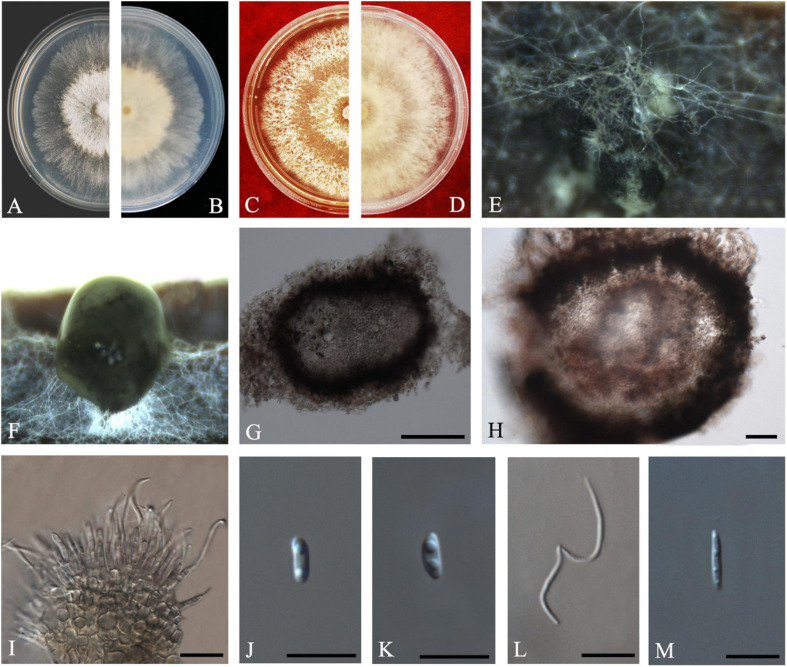
*Diaporthe masirevicii* (ZHKUCC 20-0009). **(A.B)** Colonies on PDA after 4 days at 25°C, **(C,D)** Colonies on MEA after 30 days at 25°C, **(E,F)** Colonies on MEA after 14 days at 25°C, **(G)** Conidiomata sporulating on PNA, **(H)** Pycnidium, **(I)** Conidiogenous cells, **(J,K)** Alpha conidia, **(L)** Beta conidia, and **(M)** Gamma conidia. Scale bars: **(G,H)** = 100 μm; **(I–M)** = 10 μm.

R.G. Shivas, L. Morin, S.M. Thompson and Y.P. Tan, *Persoonia* 35: 45 (2015) *Index Fungorum number*: IF808671, *Faces of fungi*: FoF 08408 (Figure 9).

*Endophytic* on *C. grandis* “Tomentosa” twigs. **Sexual morph:** not observed. **Asexual morph:** *Pycnidia* 156–568 μm × 120–316 μm ($\bar{x}$ = 256 ± 88 μm × 201 ± 51 μm), small, solitary, subglobose, conidia produced in subhyaline to brownish green drops. *Conidiophores* 9–36 μm × 1–4 μm ($\bar{x}$ = 19 ± 6 μm × 2 ± 0.6 μm), subcylindrical or filiform, unbranched. *Alpha conidia* 6–10 μm × 2–4 μm ($\bar{x}$ = 8 ± 1 μm × 3 ± 0.3 μm), cylindrical to ellipsoid, some with one end rounded and the others acute, hyaline, aseptate, biguttulate. *Beta conidia* 15–26 μm × 1–2 μm ($\bar{x}$ = 21 ± 3 μm × 2 ± 0.3 μm), filiform, hyaline, tapering toward both ends, hamate or curved. *Gamma conidia* hyaline, multiguttulate. Beta conidia are much more than the alpha conidia in the PDA.

*Culture Characteristics*: Colonies in PDA reach 85 mm diam. after 5 days on at 25°C. White aerial mycelium, irregular, undulate margin, develop concentric rings of pycnidia. Reverse white, and later become dark gray from the center.

*Material examined*: CHINA, Guangdong Province, Huazhou, isolated from healthy fruits and healthy twigs of *C. grandis* cv. “Tomentosa,” May 2019, ZY Dong and YX Shu, (dried culture ZHKU 20-0007–9); living culture ZHKUCC 20-0007–9).

*Habitat and host*: *Arachis hypogaea, Camellia sinensis, Chrysanthemoides monilifera, Chrysanthemoides monilifera subsp. rotundata, Gloriosa superba, Glycine max, Helianthus annuus, Physalis peruviana*, and *Zea mays* (Farr and Rossman, 2020).

*Known distribution*: Australia (Thompson et al., 2015; Dissanayake et al., 2017b; Yang et al., 2018b), Brazil (Pazdiora et al., 2018), China (Gao et al., 2017; Yang et al., 2020), India (Naveen et al., 2018).

*Note:* Three isolates obtained in the present study clustered together with *Diaporthe masirevicii* (BRIP 57892a) with 94% maximum likelihood, 92% maximum parsimony bootstrap, and 1.0 Bayesian posterior probability values. Morphologically these strains produce beta and gamma conidia with similar lengths of those of the *Diaporthe masirevicii* type description (Thompson et al., 2015). *Diaporthe masirevicii* was introduced by Thompson et al. (2015) as a species associated with cankers or dead trees of *Chrysanthemoides monilifera* subsp. *Rotundata, Glycine max*, *Helianthus annuus*, and *Zea mays*. To our knowledge, this is the first report of *D. masirevicii* on *C. grandis* cv. “Tomentosa” (Farr and Rossman, 2020).

#### Diaporthe passifloricola

**FIGURE 10:**
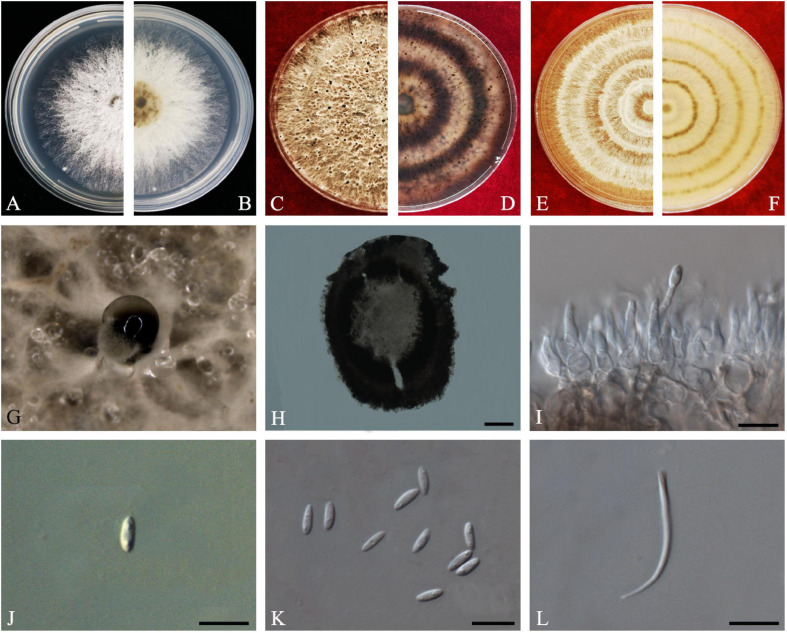
*Diaporthe passifloricola* (ZHKUCC 20-0017). **(A,B)** Colonies on PDA after 4 days at 25°C, **(C,D)** Colonies on PDA after 30 days at 25°C, **(E,F)** Colonies on MEA after 14 days at 25°C, **(G)** Conidiomata sporulating on PDA, **(H)** Pycnidium, **(I)** Conidiogenous cells, **(J,K)** Alpha conidia, and **(L)** Beta conidia. Scale bars: **(H)** = 100 μm; **(I–L)** = 10 μm.

Crous & M.J. Wingf., in Crous et al., Persoonia 36: 395 (2016) *Index Fungorum number*: IF817057, *Faces of fungi*: FoF 08411 (Figure 10).

Endophytic on *C. grandis* “Tomentosa” fruits and twigs. **Sexual morph:** not observed. **Asexual morph:** *Pycnidia* 107–388 μm × 90–256 μm ($\bar{x}$ = 228 ± 73 μm × 170 ± 53 μm), pycnidial, black, globose, exuding gray to black creamy droplets. *Conidiophores* 9–35 μm × 2–3 μm ($\bar{x}$ = 17 ± 5 μm × 2 ± 0.4 μm), hyaline, smooth, 2–3-septate, branched, densely aggregated, cylindrical. *Alpha conidia* 6–7 μm × 2–3 μm ($\bar{x}$ = 7 ± 0.3 μm × 3 ± 0.2 μm), aseptate, hyaline, smooth, cylindrical, some with one end rounded and the others acute, with biguttulate. *Beta conidia* 18–26 μm × 1–2 μm ($\bar{x}$ = 21 ± 2 μm × 2 ± 0.2 μm), smooth, hyaline, aseptate, spindle-shaped, apex acutely rounded, base truncate.

*Culture Characteristics*: Colonies on PDA reach 85 mm diam. on PDA at 25°C after 7 days. White, fluffy, aerial mycelium, and filiform margins. Reverse white, and later become yellow-brown to ochreous from the center.

*Material examined*: CHINA, Guangdong Province, Huazhou, isolated from healthy fruits and twigs of *C. grandis* cv. “Tomentosa,” May 2019, ZY Dong and YX Shu, (dried cultures ZHKU 20-0017–24); living cultures ZHKUCC 20-0017–24.

*Habitat and host*: *Passiflora foetida* (Crous et al., 2016).

*Known distribution*: China (this study) and Malaysia (Crous et al., 2016).

*Note*: In the combined multigene phylogenetic analysis of ITS, *tef1*, *tub2*, and *cal*, eight strains isolated in this study developed a well-supported clade with the *D. passifloricola* (CPC 27480) with 96% ML, 96% MP bootstrap and 1.0 BYPP values. In comparison between *D. passifloricola* and strains in the present study, they share morphologically similar characters as given in Crous et al. (2016). However, isolates obtained in this study have faster growth rate on PDA and radial margins. In addition, comparisons of gene regions between isolates from this study and *D. passifloricola* type, there is a 1.4% nucleotide difference in ITS with 555 nucleotides. In protein-coding regions, 0.55% differences in *tub2* (547 nucleotides). This is the first record of *D. passifloricola* on *C. grandis* cv. “Tomentosa” (Farr and Rossman, 2020).

#### Diaporthe perseae (Zerova)

**FIGURE 11:**
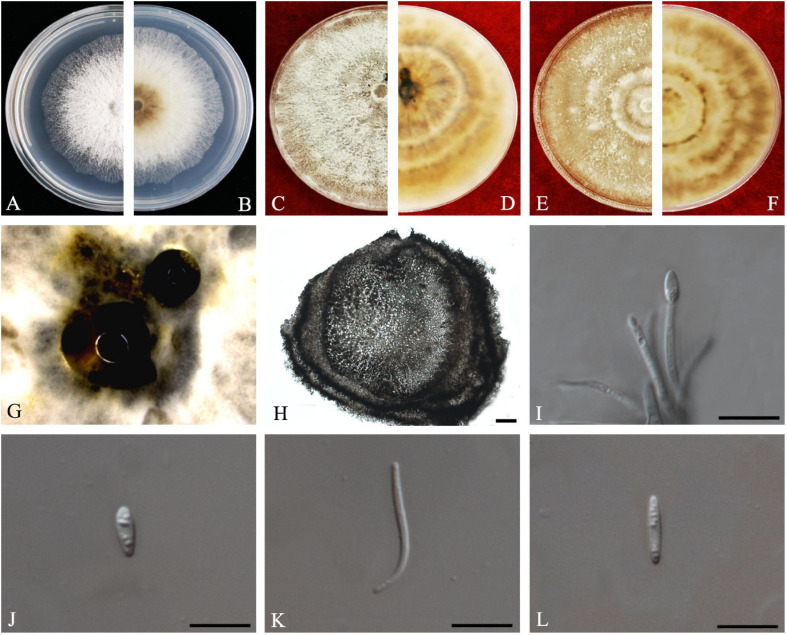
*Diaporthe perseae* (ZHKUCC 20-0010). **(A,B)** Colonies on PDA after 3 days at 25°C, **(C,D)** Colonies on PDA after 30 days at 25°C, **(E,F)** Colonies on MEA after 14 days at 25°C, **(G)** Conidiomata sporulating on PNA. **(H)** Pycnidium, **(I)** Conidiogenous cells, **(J,K)** Alpha conidia, and **(L)** Beta conidia. Scale bars: **(H)** = 100 μm; **(I–L)** = 10 μm.

R.R. Gomes, Glienke and Crous, *Persoonia* 31: 29 (2013) *Index Fungorum number*: IF802944, *Faces of fungi*: FoF 08695 (Figure 11).

*Endophytic* on *C. grandis* “Tomentosa” leaves. **Sexual morph:** not observed. **Asexual morph:** *Pycnidia* 187–674 μm × 141–484 μm ($\bar{x}$ = 394 ± 142 μm × 280 ± 94 μm), subglobose or lageniform, conidial drops yellowish, white and or transparent creamy. *Conidiophores* 10–28 μm × 2–3 μm ($\bar{x}$ = 16 ± 4 μm × 2 ± 0.5 μm), 1–3-septate, hyaline, smooth, cylindrical to sinuous, branched, and densely aggregated straight. *Alpha conidia* 5–8 μm × 2–3 μm ($\bar{x}$ = 7 ± 1 μm × 2 ± 0.2 μm), aseptate, hyaline, smooth, fusoid to ellipsoid, tapering toward both ends, straight, apex subobtuse, base subtruncate, with two to four guttules. *Beta conidia* 17–28 μm × 1–2 μm ($\bar{x}$ = 24 ± 3 μm × 1 ± 0.2 μm), aseptate, hyaline, spindle-shaped, smooth, apex acutely rounded, base truncate.

*Culture Characteristics*: Colonies on PDA at 25°C reach 85 mm diam. after 4 days. White and later turns pale white with patches of sienna, filamentous, entire margin. Reverse white, and with age produce umber color patches turning into sienna.

*Material examined*: CHINA, Guangdong Province, Huazhou, isolated from healthy leaves of *C. grandis* cv. “Tomentosa,” May 2019, ZY Dong and YX Shu, (dried culture ZHKU 20-0010); living culture ZHKUCC 20-0010).

*Habitat and host*: *Persea gratissima* (Gomes et al., 2013), *Mangifera indica* (Lim et al., 2019).

*Known distribution*: Malaysia (Lim et al., 2019), Netherlands (Gomes et al., 2013) China (this study).

Note: A single isolate from the present study clustered together with the *Diaporthe perseae* (CBS 151.73) with 73% maximum likelihood, 72% maximum parsimony bootstrap, and 1.0 bayesian posterior probability values. Morphologically this strain produces a similar length of both alpha and beta conidia to the *Diaporthe perseae* (Gomes et al., 2013). *Diaporthe perseae* was introduced by Gomes et al. (2013) as a species associated with branches of dying *Persea gratissima*. This is the first report of *D. perseae* on *C. grandis* (Farr and Rossman, 2020).

#### Diaporthe sennae

**FIGURE 12:**
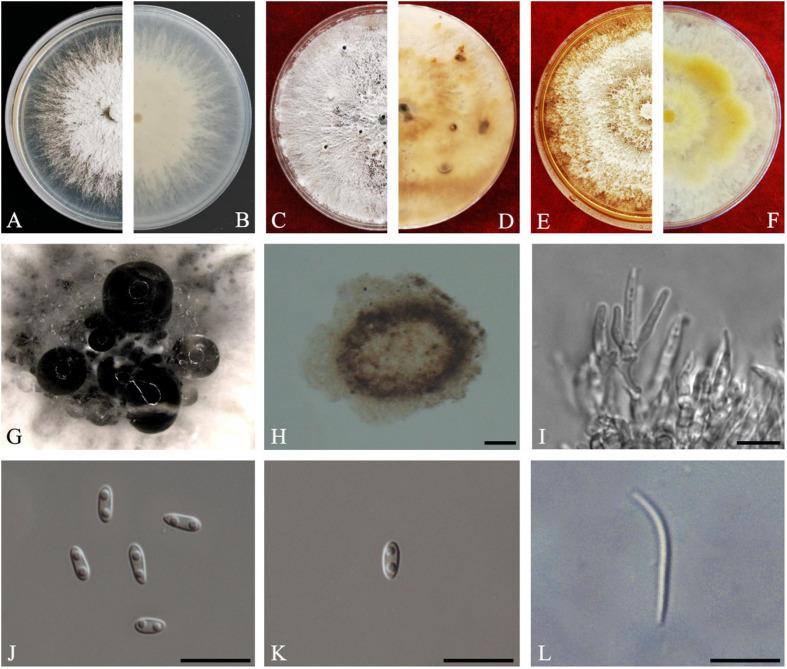
*Diaporthe sennae* (ZHKUCC 20-0011). **(A,B)** Colonies on PDA after 4 days at 25°C, **(C,D)** Colonies on PDA after 30 days at 25°C, **(E,F)** Colonies on MEA after 14 days at 25°C, **(G)** Conidiomata sporulating on PDA, **(H)** Pycnidium, **(I)** Conidiogenous cells, **(J)** Alpha conidia, **(K)** Beta conidia, and **(L)** Gamma conidia. Scale bars: **(I)** = 100 μm; **(J–L)** = 10 μm.

C.M. Tian and Qin Yang, in Yang, Fan, Du and Tian, Phytotaxa 302(2): 149 (2017) *Index Fungorum number*: IF820452, *Faces of fungi*: FoF08696 (Figure 12).

*Endophytic* on *C. grandis* “Tomentosa” leaves. **Sexual morph:** not observed. **Asexual morph:** *Pycnidial, Pycnidia* 149–756 μm × 95–692 μm ($\bar{x}$ = 349 ± 136 μm × 281 ± 126 μm), subglobose or lageniform, scattered, erumpent through the bark surface. *Conidiophores* 16–34 μm × 1–3 μm ($\bar{x}$ = 23 ± 5 μm × 2 ± 0.4 μm), cylindrical, hyaline. *Alpha conidia* 6–8 μm × 3–3 μm ($\bar{x}$ = 7 ± 1 μm × 3 ± 0.2 μm), cylindrical to ellipsoid, hyaline, aseptate, smooth, multiguttulate, usually two guttules at each end. *Gamma conidia* cylindrical, hyaline, multiguttulate. *Beta conidia* 18–36 μm × 1–2 μm ($\bar{x}$ = 26 ± 4 μm × 2 ± 0.2 μm), hyaline, aseptate, straight to hamate.

*Culture Characteristics*: After 5 days on PDA, colonies reach 85 mm diam. at 25°C. White, radial hyphal growth, becoming gray-white, develop pycnidia after 7 days. Reverse white, then pale brown from the center and forming concentric rings of pycnidia.

*Material examined*: CHINA, Guangdong Province, Huazhou, isolated from a healthy fruit of *C. grandis* cv. “Tomentosa,” May 2019, ZY Dong and YX Shu, (dried culture ZHKU 20-0011); living culture ZHKUCC 20-0011).

*Habitat and host*: *Senna bicapsularis* (Yang et al., 2017a).

*Known distribution*: China (Yang et al., 2017a).

*Note*: The ZHKUCC 20-0011 isolate obtained in this study clustered together with the *D. sennae* (CFCC 51636) with 95% maximum likelihood, 92% maximum parsimony bootstrap, and 1.0 Bayesian posterior probability values. Morphologically the isolate in this study produces a similar size of alpha and beta conidia to the *D. sennae* type description (Yang et al., 2017a). Gamma conidia were also observed in strain ZHKUCC 20-0011 of this study after 7 days on PDA. *Diaporthe sennae* was introduced by Yang et al. (2017a) as a species associated with dieback of *Senna bicapsularis*. This is the first record of *D. sennae* on *C. grandis* cv. “Tomentosa” (Farr and Rossman, 2020).

## Discussion

In the present study, *Diaporthe* species were isolated as endophytes from *C. grandis* cv. “Tomentosa” in China with 24 isolates from fruits, leaves, and twigs. Based on the multigene phylogeny, all 24 isolates from this study were grouped in 11 distinct clades within the *Diaporthe* phylogenetic tree. Among them, two species (*D. arecae* and *D. biconispora*) are already known to be associated with *C. grandis*. Nine new host records were identified namely: *D. apiculata, D. aquatica, D. limonicola, D. masirevicii, D. passifloricola, D. perseae*, and *D. sennae.* The remaining two species were identified as novel and introduced here as *D. endocitricola*, and *D. guangdongensis*. This study is the first comprehensive analysis of endophytic fungi associated with *C. grandis* in China.

The host species in this study is *C. grandis* cv. “Tomentosa,” which is commonly known as “huajuhong” in China is a famous traditional Chinese medicinal plant, which has been used to alleviate cough and phlegm for several hundred years (Jiang et al., 2014). It has been proved that the endophytic fungi associated with medicinal plants have the ability to act as biological control agents (Carvalho et al., 2012) and some have anticancer activities (Carvalho et al., 2012). A few studies have revealed that secondary metabolites produced by endophytic fungi could be novel sources of medicinal compounds (Strobel et al., 2001; Kusari et al., 2012). Therefore, further studies are necessary to understand the relationship between medicinal properties and a plant’s endophytic biota.

In the present study, endophytes were isolated from fruits, leaves, and twigs. *Diaporthe masirevicii* and *D. passifloricola* were isolated from fruits and twigs of *C. grandis*, while the other species were isolated from only one tissue type. A lower degree of colonization was observed on leaves (the only two species isolated were; *Diaporthe perseae* and *Diaporthe biconispora*). Similar to this study, Gond et al. (2007) and Huang et al. (2015) observed a lower number of endophytic species in leaves of *Citrus* spp. One endophytic species might occur in different tissues in the same host (Huang et al., 2015). However, the endophytic colonization in different tissues of the same plant might also vary (Taylor et al., 1999; Huang et al., 2015). For example, greater numbers of endophytic fungi were isolated from flowers and seeds than that from vegetative organs like stems and leaves (Braun et al., 2003). It has also been observed that greater numbers of endophytes could be isolated from veinal tissues than from interveinal tissues (Taylor et al., 1999). These variations might be a result of differences in the tissue organizational structure and the different nutrition content of each tissue type (Rana et al., 1997; Huang et al., 2015). However, the exact underlying reasons and mechanisms for these variations are not known. It is thus clear that further studies are needed to compare the variations in endophytic colonization according to different seasons or different stages of maturity of the plant.

*Diaporthe biconispora* was previously reported as an endophyte in branches of *Citrus sinensis* in China (Huang et al., 2015). In the present study, this species was isolated from leaves. Thus, *D. biconispora* may be a common endophytic species in Chinese *Citrus* plants. *Diaporthe limonicola* has been reported as pathogenic on *Citrus* sp. (Huang et al., 2015; Guarnaccia and Crous, 2017) and was first reported as a dieback pathogen of lemon trees in Europe. This species causes serious cankers on *Citrus limon*, *C. aurantiifolia*, *C. reticulata*, and *C. sinensis* (Guarnaccia and Crous, 2017). Identification of previously known pathogenic species as endophytes might reveal the opportunistic pathogenic nature of the *Diaporthe* species (Manawasinghe et al., 2019). Moreover, this is also important to develop quarantine measures to prevent the introduction of these species into new localities. Two of the species isolated in this study have been reported as pathogens on several other hosts. *Diaporthe masirevicii* has been reported causing peanut stem and peg dieback in Australia (Thompson et al., 2018), *Gloriosa superba* leaf blight in India (Naveen et al., 2018) and *Physalis peruviana* fruit rot in Brazil (Pazdiora et al., 2018). *Diaporthe perseae* was reported causing stem-end rot of mango (Lim et al., 2019). Therefore, further studies are necessary to understand the pathogenicity of these endophytic strains and the factors that determine their pathogenicity on *Citrus.*

In addition to previously known species from *Citrus*, in the present study, we identified nine novel host records and two new species. Novel species and host records are an indication of the ability of *Diaporthe* to evolve rapidly (Manawasinghe et al., 2019). Furthermore, this reflects the high species diversity of *Diaporthe* associated with a single host. Several studies have revealed the high species richness of *Diaporthe* species as endophytes on different hosts (Skaltsas et al., 2011; Rhoden et al., 2012). When there are diverse species associated with a single host, there is the potential of emerging new pathogens on *Citrus*. This could be a result of the species developing into taxa with greater virulence, or divergence of existing species into a novel species *via* long term exposure to natural and human-mediated factors. One possible phenomenon is the application of fungicides for other known phytopathogens while non-target fungal species become pathogenic a few years later. Thus, it might be challenging to control current disease while eliminating new disease occurrences. To overcome this, further studies are necessary to understand the interaction of endophytes with phytopathogenic genera and their effect on the *Citrus* microbiome.

## Conclusion

In the present study, eleven endophytic *Diaporthe* species were isolated and identified from *C. grandis* cv. “Tomentosa” in China. Two new species *D. endocitricola* and *D. guangdongensis* were introduced. This study reveals the existence of several previously known pathogenic *Diaporthe* species as endophytes. Thus, it reflects the opportunistic nature of *Diaporthe* species as phytopathogens. However, further studies are necessary to understand the pathogenic potential of these endophytic taxa on *C. grandis* or other *Citrus* species in China. These results will open a discussion on interactions between fungal species on a particular host as endophytes, pathogens and potential biocontrol agents. In addition, these results will provide a platform to develop antimicrobial compounds, and to understand the contribution of endophytes to the medicinal values of the plant.

## Data Availability Statement

The datasets presented in this study can be found in online repositories. The names of the repository/repositories and accession number(s) can be found in the article/Supplementary Material.

## Author Contributions

ZD and ML conceived the research and planned the basic research. YS provided the materials. YS, YH, and ML conducted the experiments. ZD, ML, and IM prepared the manuscript. ZD, ML, IM, and AP analyzed the data. AP, KH, AD, and MX revised the manuscript. All authors read and approved the final manuscript.

## Conflict of Interest

The authors declare that the research was conducted in the absence of any commercial or financial relationships that could be construed as a potential conflict of interest.

## Footnotes

1 http://www.ebi.ac.uk/Tools/msa/mafft/

2 http://purl.org/phylo/treebase/phylows/study/TB2:S26384?x-access-code=e34e24939299e00766a5bf45970dabb0&format=html

3 www.indexfungorum.org
